# Dynamics of nuclear receptor gene expression during Pacific oyster development

**DOI:** 10.1186/s12861-016-0129-6

**Published:** 2016-09-29

**Authors:** Susanne Vogeler, Tim P. Bean, Brett P. Lyons, Tamara S. Galloway

**Affiliations:** 1 School of Biosciences, College of Life and Environmental Sciences, University of Exeter, Stocker Road, Exeter, EX4 4QD UK; 2 Centre for Environment, Fisheries and Aquaculture Science, Cefas Weymouth Laboratory, Barrack Road, Weymouth, DT4 8UB UK

**Keywords:** Nuclear receptors, Gene expression, Development, *Crassostrea gigas*, Invertebrates

## Abstract

**Background:**

Nuclear receptors are a highly conserved set of ligand binding transcription factors, with essential roles regulating aspects of vertebrate and invertebrate biology alike. Current understanding of nuclear receptor regulated gene expression in invertebrates remains sparse, limiting our ability to elucidate gene function and the conservation of developmental processes across phyla. Here, we studied nuclear receptor expression in the early life stages of the Pacific oyster, *Crassostrea gigas*, to identify at which specific key stages nuclear receptors are expressed

**Results:**

We used quantitative RT-PCR to determine the expression profiles of 34 nuclear receptors, revealing three developmental key stages, during which nuclear receptor expression is dynamically regulated: embryogenesis, mid development from gastrulation to trochophore larva, and late larval development prior to metamorphosis. Clustering of nuclear receptor expression patterns demonstrated that transcriptional regulation was not directly related to gene phylogeny, suggesting closely related genes may have distinct functions. Expression of gene homologs of vertebrate retinoid receptors suggests participation in organogenesis and shell-formation, as they are highly expressed at the gastrulation and trochophore larval initial shell formation stages. The ecdysone receptor homolog showed high expression just before larval settlement, suggesting a potential role in metamorphosis.

**Conclusion:**

Throughout early oyster development nuclear receptors exhibited highly dynamic expression profiles, which were not confined by gene phylogeny. These results provide fundamental information on the presence of nuclear receptors during key developmental stages, which aids elucidation of their function in the developmental process. This understanding is essential as ligand sensing nuclear receptors can be disrupted by xenobiotics, a mode of action through which anthropogenic environmental pollutants have been found to mediate effects.

**Electronic supplementary material:**

The online version of this article (doi:10.1186/s12861-016-0129-6) contains supplementary material, which is available to authorized users.

## Background

Nuclear receptors (NRs) are one of the largest classes of transcription factors in metazoan species and regulate many cellular functions through manipulation of gene expression. Although NRs are present even in the simplest animals in low numbers (demosponge: 2 NRs [1]), extensive diversification of NR families has occurred in Bilateria through gene duplications, gene loss and diversification [1–4]. In vertebrate species, NRs are essential for regulating gene expression during complex processes, in particular during development, which is one of the most dynamic periods of NR activity [5, 6]. For invertebrates, however, information on NR regulated gene expression is limited. Nevertheless, many developmental processes in metazoans are highly conserved and similar features are shared among diverse phyla. Understanding the presence and participation of NRs in phases of tightly controlled gene expression, particularly during developmental stages, is highly desirable. Not only is this a requirement for understanding the intrinsic biology, but also because alteration of NR function is one of the key routes through which normal biology can be disrupted by external factors, often resulting in abnormal phenotypes.

Typical NRs consists of five different domains, which include the highly conserved C domain, also denoted as the DNA-binding domain (DBD), and the moderately conserved ligand binding (E-) domain (LBD). The high sequence conservation of the DBD and LBD in particular, allows for phylogenetic classification of NR subfamilies (NR0-NR6) and their constituent subgroups [7, 8]. NRs regulate gene expression by attaching the DBD to specific response elements in the promoter of target genes, with the whole protein structure functioning as monomer, homodimer or heterodimer [9, 10].

One of the most interesting characteristics of NRs is their capability to interact with endogenous or exogenous compounds through ligand binding, a feature which has been described for a subset of NRs, such as for hormonal (steroid and thyroid hormones) and retinoid regulated receptors. In addition, other NRs do not require any ligand binding and function in a constitutively activated manner [9]. Ligand-binding NRs are able to bind to exogenous compounds. When present in high doses or as mimics of natural ligands, these xenobiotics can lead to disruption of normal NR function [11]. Xenobiotics in the environment are commonly introduced by anthropogenic pollution and can affect various developmental and physiological processes in humans and wildlife [12, 13]. In many fish species exposure to xenoestrogens such as bisphenol A (BPA), 17α-ethinylestradiol and dichlorodiphenyltrichloroethane, have caused developmental malformations and had negative effects on reproduction [12, 14, 15]. Many of the effects of environmental estrogens are known to be mediated through interaction with NRs [15–18]. Tributyltin (TBT), an organotin and an additive in antifouling paints for boats, has been shown to cause imposex, (superimposition of male organs on females) in > 20 gastropod species [19] as well as developmental failure and reproduction impairment in bivalves [20–23]. TBT has been identified as a xenobiotic ligand for vertebrate and gastropod NRs [24–26] and a link between NRs and TBT-mediated disruption has been proposed [26–28]. As the expression of NRs varies between different life stages, the response of an animal to a xenobiotic can vary according to the life stage.

The function and presence of NRs in development, reproduction and homeostasis in vertebrate species is well studied, but knowledge of receptor participation in invertebrate systems has been less well investigated. Previously, we [29] reported the presence of 43 NRs in the Pacific oyster, *Crassostrea gigas,* and described their phylogenetic relationship to other known NR homologs in human and *Drosophila*. The Pacific oyster is a bivalve species (Clade: Lophotrochozoa, Phylum: Mollusca) and as a sessile filter feeder, it is a commonly used organism for biological monitoring [30–32]. Oysters live along coasts and estuaries worldwide and are under constant anthropogenic pressure including from industrial, agriculture and sewage pollution. Although oyster development has been well studied due to the high economic interests in aquaculture for food, the underlying molecular mechanisms of gene regulation during development remain mostly unknown. Here, we provided an overview of the presence of NRs in Pacific oyster life stages, including the early embryo and larva. We studied 34 of the 43 NRs genes, for which expression could be verified by quantitative RT-PCR (qPCR), and assessed the expression patterns for phylogenetically related groups of genes across early life stages, also including a comparison with adults of both sexes. We discuss the results in the context of the three identified developmental stages (early, mid and late development) and the potential participation of NR genes in embryonic development and sexual differentiation. In particular we focused on those receptors which have previously shown a potential for xenobiotic disruption in other species; the retinoid X receptor (RXR), retinoic acid receptor (RAR), thyroid receptor (THR), estrogen receptor (ER), estrogen-related receptor (ERR), peroxisome proliferator-activated receptor (PPAR), ecdysone receptor (EcR), and xenobiotic-sensing receptor subfamily group NR1J.

## Results

**Fig. 1:**
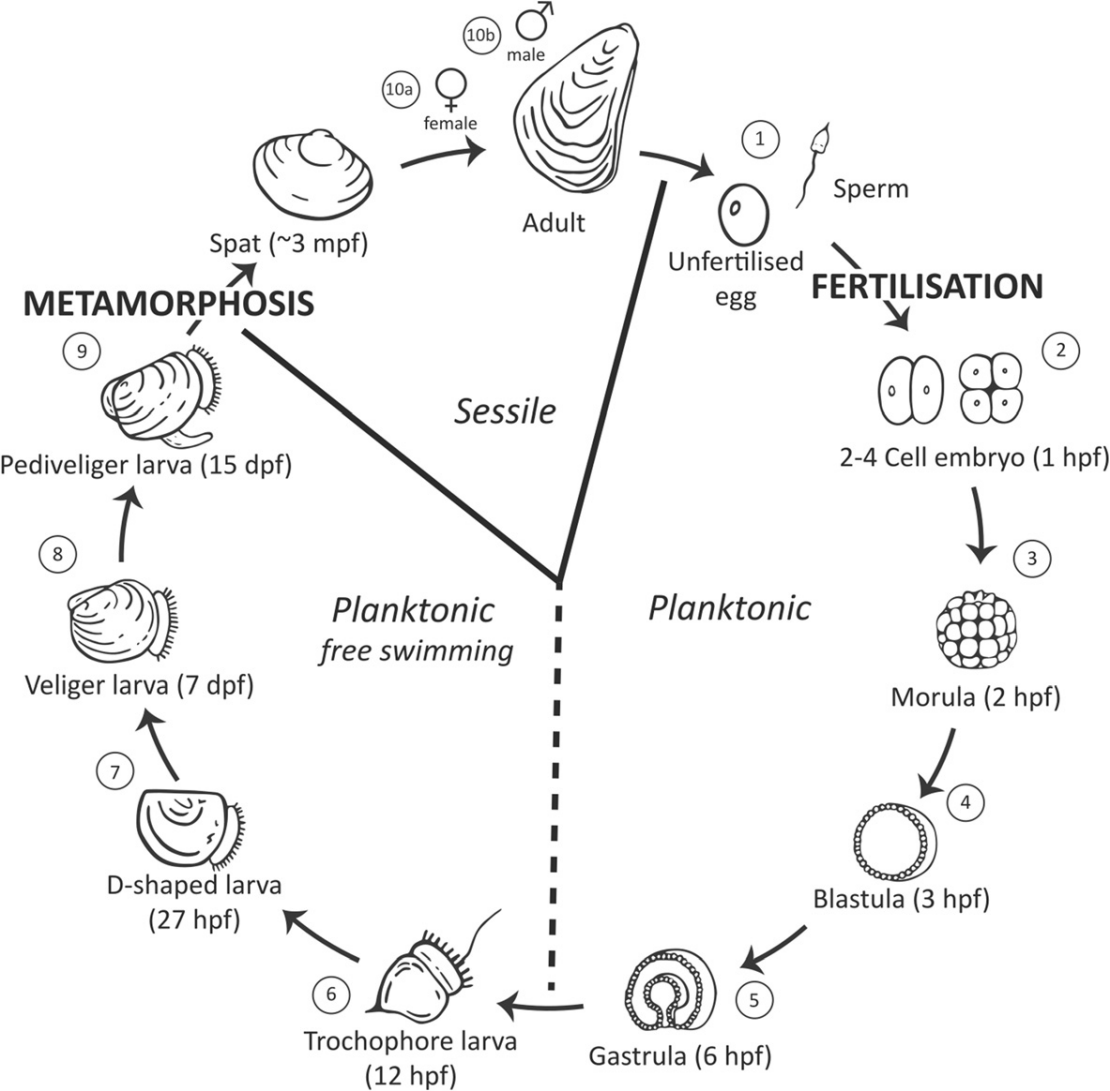
Life cycle of the Pacific oyster, *Crassostrea gigas*. Numbers 1-10 represent sampling points for nuclear receptor expression analysis. hpf: hours post fertilisation; dpf: days post fertilisation; mpf: month post fertilisation

**Fig. 2:**
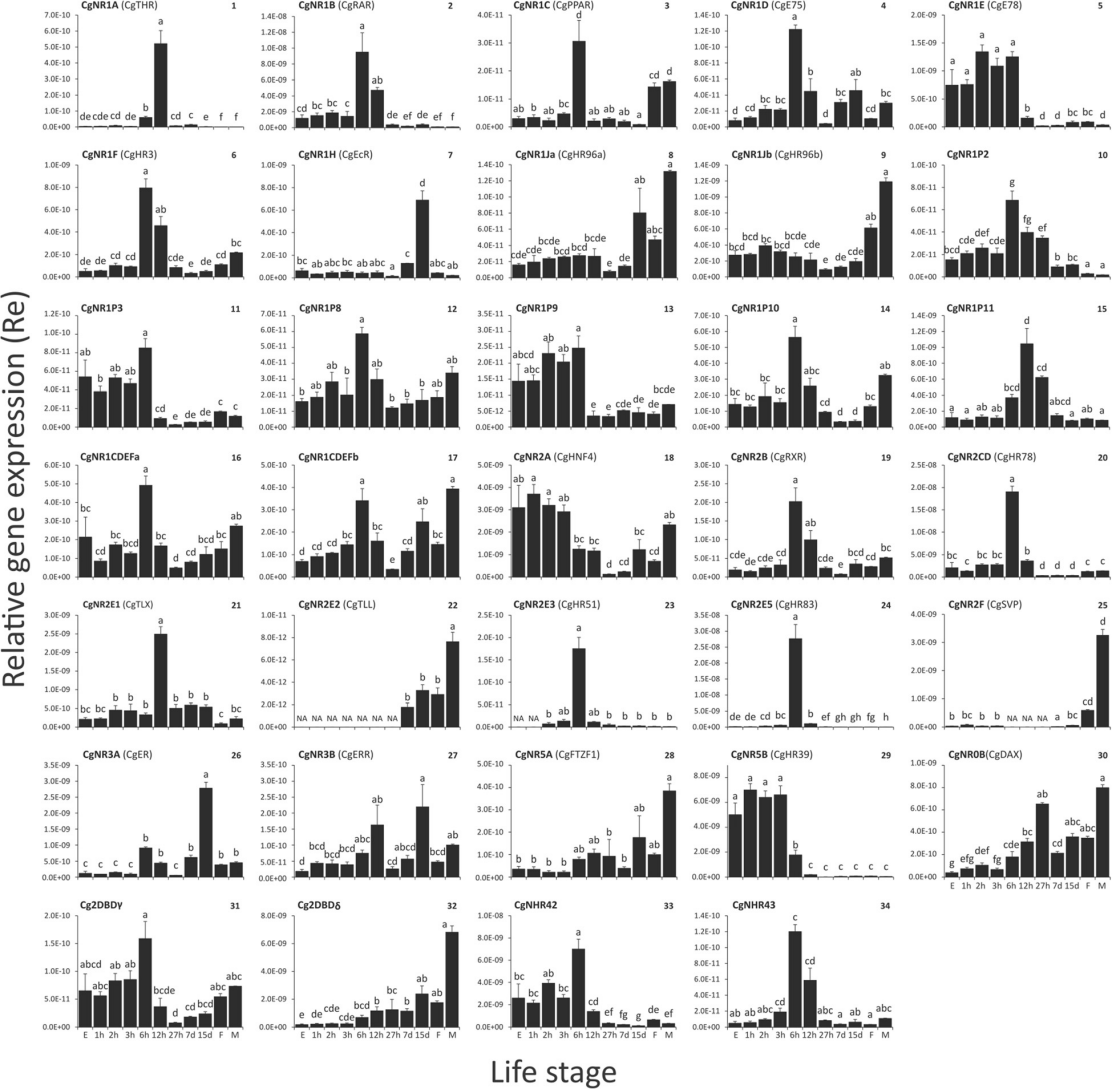
Relative gene expression of 34 *Crassostrea gigas* nuclear receptors in different life stages. Gene expression was measured using quantitative RT-PCR. Relative gene expression was calculated using a normalisation factor computed with the three reference genes and statistically analysed as described in the methods section. Alternative names for oyster nuclear receptors based on their closest identified homologs in *Homo sapiens* or *Drosophila melanogaster* [13], are provided in brackets. Bars indicate the mean ± standard error of three independent measurements per time point. Different letters above each bar represent groups that were significantly different (*p* = 0.05); same letters: no significant difference. N/A: expression below detection limit; h: hour post fertilisation; d: days post fertilisation; E: unfertilised eggs; F: female; M: male

Quantitative RT-PCR was used to measure the expression of 34 NRs in ten different life stages of the Pacific oyster, including nine developmental stages, and male and female adult individuals (Fig. 1). In general, NR expression was measured during all life points and showed variation between developmental stages (Fig. 2). A few NR transcripts (CgNR2E2, CgNR2E3 and CgNR2F), were below the limit of detection (N/A) at some life stages. Expression of each receptor was calculated relative to a normalisation factor derived from three reference genes. Elongation factor-1 α (EF-1), ribosomal protein S18 (RS18) and ribosomal protein L7 (RL7) were verified as the most suitable reference genes among other commonly used housekeeping genes (glyceraldehyde-3-phosphate dehydrogenase, glutathione S-transferase, α-tubulin) by the programme geNorm v3 (Additional file 1).

**Fig. 3:**
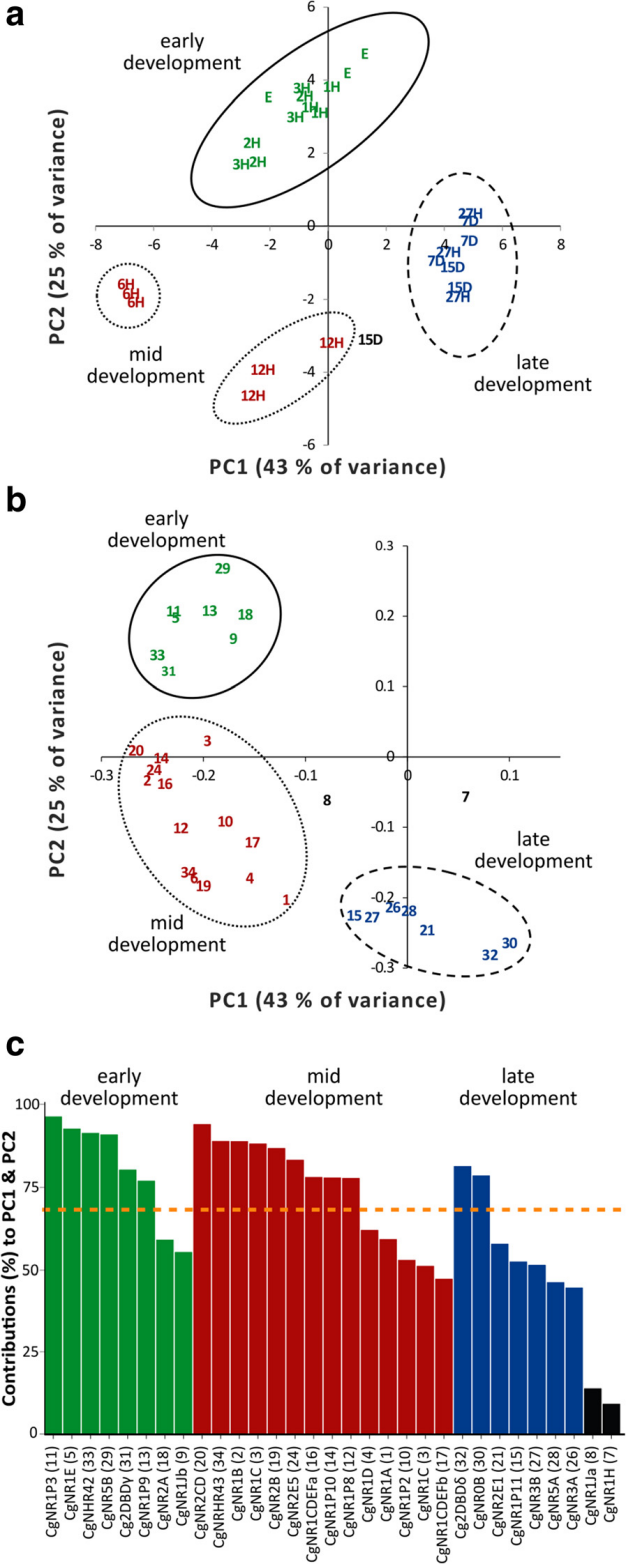
Principle component analysis (PCA) of 31 *Crassostrea gigas* nuclear receptor gene expression data. **a** Scatterplot of the first two PCA components of developmental stages indicating separation based upon the variance observed in the expression levels of 31 of the 34 nuclear receptor genes. Principal component 1 (PC1) and 2 (PC2) explain 43 and 25 % of variance, respectively. **b** Scatterplot of PC1 and PC2 scores indicating the separation of 31 nuclear receptors based on expression across life stages. Circles around measurements represent distinct clustering for developmental stages or nuclear receptors: early development (*solid line*), mid development (*dotted lines*), late development (*dashed line*). Number codes for nuclear receptors can be found in (**c**). **c** Bar chart showing the contribution (in percentage) of each nuclear receptor towards the variability of PC1 and PC2. The orange dashed line: expected average contribution. Letters/numbers/bars: early development (*green*), mid development (*red*), late development (*blue*), receptors not assigned (*black*). h: hour post fertilisation; d: days post fertilisation; E: unfertilised eggs

Principal component analysis (PCA) for developmental stages (excluding adult stages) (Fig. 3) was conducted for 31 of 34 NRs, excluding those receptors which showed an expression below detection limit. Principal component 1 (PC1) accounted for 42 % of the overall variance in gene expression among developmental stages and principal component 2 (PC2) accounted for 25 % of the overall variance. Based on the observed variance in the expression levels of the NRs, four distinct life events are clearly distinguishable (Fig. 3a): early embryo development (unfertilized egg – 3 hpf), mid development, divided into gastrulation (6 hpf) and trochophore larval stage (12 hpf), and late larval development (27 hfp – 15 dpf).

Similar clustering was detected by separating the NRs based on the observed variance in different life stages (Fig. 3b). Three clusters of NRs emerged and could be categorised in three of the four previously detected key stages: early, mid and late development. Within the mid development group, the gastrulation (6 hpf) and trochophore larval (12 hpf) stages were not as clearly distinguished as they had been during the previous observation of the variance in the expression levels of NRs. Most of the NRs in the detected early and mid development stages contribute to the observed principle components (Fig. 3c): early development: CgNR1P3, CgNR1E, CgNHR42, CgNR5B, Cg2DBDγ; CgNR1P9; mid-development: CgNR2CD, CgNHR43, CgNR1B, CgNR1F, CgNR2B, CgNR2E5, CgNR1CDEFa, CgNR1P10, CgNR1P8. Only two NRs (Cg2DBDδ, CgNR0B) display a contribution higher than the expected average contribution for the late development stage. CgNR1H and CgNR1Ja could not be clearly assigned to one of the three key developmental stages by the PCA (Fig. 3) and did not contribute towards the observed variance in different developmental life stages (Fig. 3c). The expression profile of CgNR1H (Fig. 2) showed an increase at 15 dpf compared to all other developmental and adult stages. CgNR1Ja, showed a comparable expression pattern to its closest oyster receptor paralog CgNR1Jb, which assigned with the NR group expressed during early development. The moderate expressions in early and mid development of both receptors are replaced by low expression during later development and high expression in adult life stages. In addition, CgNR1Ja showed a high expression peak at 15 dpf.

Previously we identified a novel subfamily group, NR1P, in the pacific oyster comprising 11 NRs [29]. The expressions of six of these receptors (NR1P2, NR1P3, CgNR1P8, CgNR1P9, CgNR1P10, CgNR1P11) were analysed and they all display differences in their expression profiles among different life stages (Fig. 3b): two receptors (NR1P3(11), NR1P9(13)) are mainly expressed at early development; three at mid-development (NR1P2(10), NR1P8(12), NR1P10(14)); and one at late development (NR1P11(15)).

The sex of adults was determined by visually observing the presence of developed oocytes (eggs) or spermatozoa in the gonads. Gene expression data (Fig. 2) shows a difference in expression patterns between unfertilized eggs and adult females. Therefore, we assume that the expression measured in female adults, which were at the beginning of the gametogenesis, was not entirely due to the presence of eggs. The same is assumed for males as RNA concentration in sperm is very low [33].

**Fig. 4:**
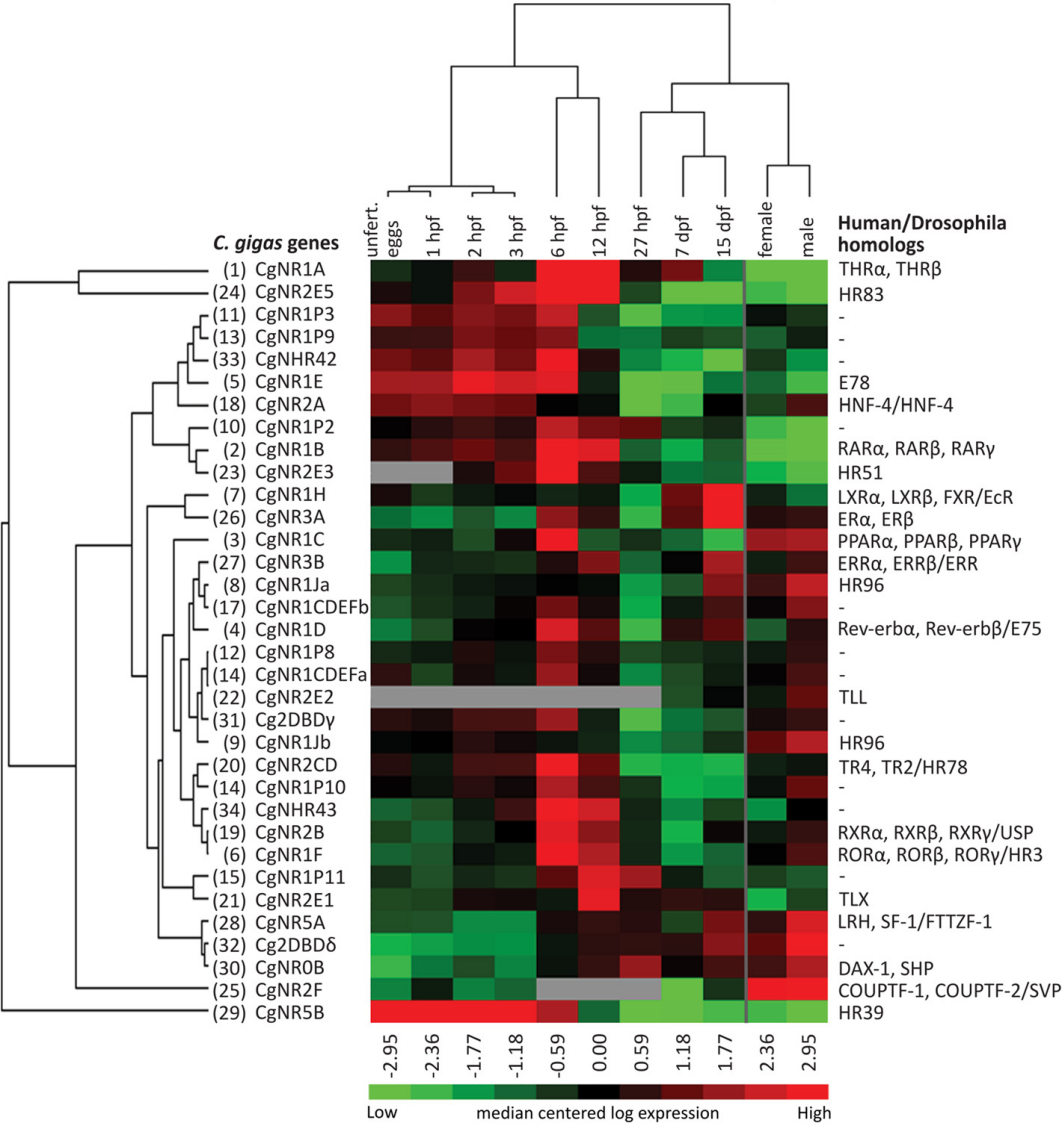
Heat map of all 34 *Crassostrea gigas* nuclear receptor gene expression data among different life stages. The mean of the relative expression of the three biological replicates were log transformed and centred. Cladogram of nuclear receptors (*left*) and of life stages (*above*) indicate groups with similar expression pattern. *C. gigas* nuclear receptor homologs in human and/or *Drosophila melanogaster* are provided in a table next to it. Numbers in brackets: Number codes for nuclear receptors accordant to Fig. 2. Grey boxes: expression below detection limit. hpf: hours post fertilisation; dpf: days post fertilisation

Individual analyses of adults (Fig. 2) show equal levels of expression for most males and females, with only five NRs showing significant differences between sexes. An additional PCA was conducted (Additional file 2) to identify general expression patterns between males and females among developmental and adult stages. Males and females vary in their NR expression and each sex also separates from most of the developmental stages (likenesses to pediveliger stage (15 dpf)). The cladogram of a heat map analysis of all NRs (Fig. 4) shows a similar separation of adults and developmental stages, in particular to early and mid developmental stages.

The clustering of the gene expression analysis does not correspond with the phylogenetic clustering of the NR subfamily groups, which also shows divergent temporal expression patterns during development. This becomes particularly apparent in the heat map analysis of all NR expressions (Fig. 4), in which all developmental stages and both adult life stages have been included. The heat map shows comparable results to the PCA; three main developmental stages, early, mid and late development, plus a further adult life stage were identified with hierarchical clustering of the life stages. Clustering of NRs based on their expression profile does not concur with the phylogenetic relationship of these NRs. The dendogram of the NR hierarchical clustering shows no coherence between phylogenetically related NRs. For example, during early development (unfertilised eggs – 3hpf), receptors belonging to subfamily 1 (e.g., 11, 13, 5, 10, 2) group together with receptors member of subfamily 2 (e.g., 18, 23) or non-assigned receptors (e.g., 33) (Fig. 4).

## Discussion

We show here that the Pacific oyster, like many other complex multicellular metazoans, differentially expresses a variety of NRs during its lifetime. The expression pattern analysis of 34 NRs throughout nine developmental time points clusters into three distinct life stages, showing dynamic changes in receptor expression: an early, a mid and a late developmental stage. Observationally, the mid developmental stage can be further divided into two separate developmental processes: gastrulation and trochophore larval stages, which include organ differentiation and shell development. Male and female adult life stages show an overall separation from the other developmental stages as well as from each other. However, the individual analysis of male and female expression patterns demonstrates only a few cases for which NRs are differentially expressed. The observed clustering of NR expression does not correlate with each receptor’s phylogenetic relationship, and belonging to the same subfamily does not result in similar patterns of expression, indicating differential or redundant functions. The members of the novel subfamily group NR1P also show differences in their expression among each life stage, which suggests that these receptors fulfil different functions, irrespective of their close phylogenetic relationship.

### Early development: embryogenesis

Embryogenesis, defines the first few steps in the life of a freshly fertilised egg in metazoans. During this event, RNA gene transcripts are thought to be maternal or zygotic in origin: maternally synthesized RNAs and proteins are stocked in oocytes during female gametogenesis [34]. A Pacific oyster zygote undergoes its first 2–4 cell division within an hour of fertilisation and is shortly after followed by the morula (2 hpf), blastula (3 hpf) and gastrula (6 hpf) stages (Fig. 1).. The expression of NRs at these early life stages did not significantly differ from the expression in unfertilised eggs for any tested NR. This suggests that the first NRs expressed during early development in oysters are provided by the mother. Nevertheless, maternal RNA is not universally stable and will degrade over time. It is replaced or diluted by zygotic gene expression, a process that is called maternal-to-zygotic transition (MZT) [34, 35]. This RNA destabilisation varies spatially and temporally, depending on gene, degradation mechanism and species, but the event of maternal RNA decay appears to be evolutionarily conserved among metazoans [36, 37]. Embryonic transcriptional activation through zygotic genome activation (ZGA) for a specific gene usually results in an increase in expression during early embryogenesis [35]. In this study, the PCA of gene expression for 34 oyster NRs did not distinguish a subgrouping within the early developmental group (Fig. 3a), which would indicate a collective ZGA onset for the majority of the NRs. The data suggests that the MTZ time schedule is not concordant for most NRs. It is unclear if and which NRs are actually regulated by ZGA in invertebrates. Although research on *Drosophila* suggests that most of the transcription factors are strictly zygotic [38], other data indicates that some transcription factors are of maternal origin and are required for ZGA onset and maternal RNA destabilisation [38–40]. Nevertheless, the resolution of this study for distinguishing between maternal/zygotic RNA is low and additional research including a higher temporal resolution is required to comprehensively identify the origin of early NR expression or proteins.

Compared to their expression level during other life stages, a few NRs showed a high expression level during early embryogenesis: CgNR2A, CgNR5B, CgNR1P9 mostly decrease in expression after 3 hpf, and CgNR1E, CgNR1P3, Cg2DBDɣ, CgNHR42 mostly decrease after 6 hpf. Assumptions of their putative function can only be made based on their closest homologs present in other species (this is a possibility for CgNR2A, CgNR1E and CgNR5B). Early development is shaped by mitotic division, germ cell layer formation and initiation of organogenesis. CgNR2A is a homolog to the hepatocyte nuclear factor 4 (HNF4) [29], a maternally transferred NR [41–43]. CgNR2A expression also suggests a maternal origin for this transcript and its high expression indicates an important role during early development. In the frog species *Xenopus laevis* HNF4 is thought to contribute to zygotic activation of a transcription factor regulating tissue specification [43–45]. Mouse HNF4 participates in regulation processes for primary endoderm development [46] and gastrulation [47], and in *Drosophila* it plays a role in gut formation [41]. Knockout experiments in insect species indicated that E78, a CgNR1E ortholog, and HR39, a CgNR5B ortholog, are required for successful early embryogenesis, beside their main functions in female reproduction [48, 49].

### Mid development: gastrulation and shell development

The mid development stage combines two of the most decisive developmental processes in the bivalve life cycle. Gastrulation (~6 hpf) is defined by three germ layers, basic body structure development and organogenesis. At the trochophore larval stage (~12 hpf), shell development is initiated and larvae become free living, using their circular ciliary bands for locomotion [50, 51]. Differentiation and development of organs and shell begins, and, in conjunction with axial patterning, requires intensive gene regulation. Many of the oyster NRs are highly expressed during gastrulation and the trochophore larval stage, indicating a participation during these pivotal developmental stages.

CgNR2B, an ortholog to vertebrate and invertebrate retinoid X receptors (RXR), is highly expressed at 6 hpf and moderately expressed at 12 hpf. Research on different molluscan species suggests participation of RXR in organogenesis and shell development. In embryos of fresh-water snails of three different gastropod species (sister-class to bivalves), exposure to 9-*cis* retinoic acid and all-*trans* retinoic acid, natural metabolites of vitamin A, caused significant eye and shell deformation. Some embryos were even developmentally arrested at the trochophore larval stage [52–54]. 9-*cis* retinoic acid was identified as a ligand of RXR in a fresh-water and a marine gastropod snail species [25, 55], which in combination with reported exposure effects in other gastropods, indicates a biological function of RXR during optic development and shell formation in snails. Additionally, exposure of developing molluscs, including oyster species, to the organotin TBT caused serious disruption of development including shell deformities, delayed growth and larval development to the point of high death rates even at low TBT concentrations [56–58]. TBT has been identified as a xenobiotic ligand for gastropod [25] and deuterostome RXRs [24, 26].

Heterodimerisation is a common feature of NRs and RXR is the preferred heterodimer partner for many species studied so far [59, 60]. CgNR1B, an ortholog to the retinoic acid receptor RAR, is mainly expressed between 6 hpf and 12 hpf and shows a similar expression pattern as CgNR2B. This provides room for speculation about a possible interaction between these receptors, a hypothesis which is supported by the findings in two gastropod species, for which gastropod RAR orthologs were observed to heterodimerise with their RXR homologs and are able to regulate gene expression in vitro [61, 62]. To what extent the oyster RAR is sensitive to xenobiotic disruption is currently unknown. In contrast to vertebrate RARs, which are highly responsive to natural and synthetic retinoic acids, molluscan RARs seem to have no ability to bind to ligands [61, 62]. More research on oyster RXR and RAR homologs could reveal links between their prominent mid developmental expression, chemical exposure and putative binding, which has been reported for their molluscan relatives. Nevertheless, retinoid metabolism in invertebrates is proposed to be partially conserved among bilaterians [63]. In vertebrates the RAR/RXR heterodimers are involved in the regulation of a diverse variety of genes contributing to organogenesis, axial patterning and neuronal differentiation [64].

Two additional oyster NRs, which are highly expressed during mid development, should also be mentioned: The first is the peroxisome proliferator-activated receptor (PPAR) homolog CgNR1C. Protostome homologs to PPARs have only been identified in one bivalve species, *C. gigas* [29], and two gastropod species, *Lottia gigantea* and *Biomphalaria glabrata,* across all of the currently studied protostomes [65], but information on their putative function or mode of action is sparse. The high expression at 6 hpf could potentially point towards a participation of CgNR1C in gastrulation supported by research on vertebrate species. In vertebrates PPARs fulfil different functions during embryo/larval development, especially during gastrulation, ranging from fat metabolism and adipocyte development [66] to cell, neural and muscular differentiation [66, 67]. TBT has also been proposed as a xenobiotic modulator of the RXR/PPARγ heterodimer in vertebrates [24, 26, 68, 69].

The second NR is CgNR1A, an ortholog to human thyroid receptors (THR) [29]. Our data demonstrates high levels of CgNR1A expression during the trochophore larval stage and lower but significant expression during gastrulation. The vertebrate THR reacts to stimuli of thyroid hormones and is a common ligand-activated NR. The thyroid, and THRs in particular, play important roles in vertebrates during organogenesis and neural development [70] and it is suggested that the thyroid signalling pathway is conserved in invertebrate protostomes [8, 71]. Exposure to BPA causes disruption of developmental, reproduction and physical processes in vertebrates and invertebrates [15]. The xenoestrogen BPA is also a known antagonist to THR in rats in vitro [72, 73].

### Late development: pre-metamorphosis

The later stages of development in bivalves and gastropods are mainly defined by growth and shell expansion as free swimming planktonic larvae. Some of the fundamental organs and the central nervous system are further defined [74], and new features appear, so called larval organs (velum for swimming and feeding, a foot for crawling, an eyespot as a light sensing organ). The duration of the free swimming stage varies depending on species and environmental cues and terminates in metamorphosis at which larvae transform to their juvenile form (spat). After a substantial re-organisation of body parts and disappearance of the larval organs, the individual attaches to a substrate and becomes sessile [50, 75]. Some NRs are expressed just before this life changing event. CgNR1H, a ortholog to the ecdysone receptor (EcR) in Ecdysozoans [29], the sister clade of Lophotrochozoans, is highly expressed at 15 dpf. In conjunction with an RXR homolog, EcR initiates and regulates life changing events such as moulting and metamorphosis in Ecdysozoans, binding to ecdysteroids [76]. After receiving an ecdysone signal the EcR/RXR homolog heterodimer initiates a transcriptional cascade of NRs, which are responsible for further gene regulation [77]. EcR is a common target for pesticides, which results in a disturbance of this cascade, leading to disruption in insect moulting [78]. The presence of an EcR ortholog in oyster pediveliger larvae raises the question whether a similar cascade in oyster individuals is activated to initiate metamorphosis and settlement. *In silico* modelling of EcR orthologs in other lopothrochozoans already suggested the possibility for the binding of EcR to ecdysone or ecdysone-like compounds outside the ecdysozoan lineage [79]. Further research on oyster EcR will be needed to investigate its putative participation in metamorphosis and their potential for xenobiotic disruption through pesticides.

The estrogen receptor (ER) and estrogen-related receptor (ERR) homologs CgNR3A and CgNR3B [29], both highly expressed at the end of the larvae stages (15 hpf), are also worth mentioning. The ERR, also present in ecdysozoans, is a proposed precursor gene of metamorphosis in insects along with many other functions in animal development [80–82]. The ER is not present in insects, but has been widely studied in fish, regulating brain development and sexual differentiation in larvae [83, 84]. The relatively high expressions of ER and ERR homologs during the mid development stage in oysters, although unresponsive to any estrogen or estrogen-like ligands [85–87], suggest these genes might be fulfilling similar functions, in a constitutive, rather than ligand dependant, manner.

### Adults: males and females

The adult life style of the Pacific oyster differs distinctly from its free-living planktonic developmental stages and requires the regulation of different genes. Being sessile, adult individuals depend completely on their surrounding environment and do not undergo any further fundamental reconstruction of their body plan. In general, our data suggests a separation of NR expression between most of the developmental stages and adult male/female individuals, indicating that different sets of NRs are switched on during the high dynamic developmental and the stationary adult stages.

Our data also indicates a difference in NR expression for male and female adults. The Pacific oyster has separate sexes and has an annual reproductive cycle, which includes the development of oocytes and spermatozoa. As a protandric species, most Pacific oyster individuals first develop as males and may change sex to female after a few annual cycles [50, 88]. In the nematode roundworm *Caenorhabditis elegans* [89], as in vertebrates [90], sex change and reproduction is directly or indirectly regulated by NRs. he oysters used in this study were at an early stage of gametogenesis for sex identification. However, the data for individual NR comparison showed only five receptors with significant differences (CgNR1E, CgNR1CDEFb, CgNR2A, CgNR2E2, CgNR2E5), of which most have not been linked to sex dimorphism in other species. Only homologs to CgNR1E in insects have been connected to female reproduction and oogenesis [49]. CgNR1E, although expressed highly in early development and less in late and adult life stages, shows a higher expression in females than in males. Expression of the ER and ERR homologs (CgNR3A and CgNR3B respectively) was at similar levels. Previous research in gastropod species showed differences in expression for male and female reproductive tissues and no differences for other tissues [85, 86, 91]. Nevertheless, gene expression was measured in whole individuals (not separated by tissue) and sex-dependent expression could still be neutralized by pooling of tissues (e.g., high expression in gonads, but low expression in mantle and vice versa).

CgNR1C, the PPAR ortholog, is highly expressed during gastrulation, but also moderately expressed in adult life stages. TBT, a known gastropod and vertebrate RXR ligand [25, 26] causes shell thickening in adult bivalve [92, 93], as well as the development of male sex organs in females (imposex) in many gastropod species [19]. There have been previous hypothesise that this is triggered through disruption of the RXR/PPAR heterodimer, since exposure to rosiglitazone, a strong PPARγ agonist for vertebrates [94], also causes imposex in gastropods [95]. Additionally, the vertebrate RXR/PPARγ heterodimer has been identified as a target for TBT, which binds to either RXR alone or both receptors [24, 26, 68, 69]. Expression of a PPAR ortholog in combination with CgNR2B in adult individuals in the Pacific oyster supports the conceivable theories of interaction between RXR and PPAR, and further adds to the theory that this may be the primary mechanism of TBT-based endocrine disruption in molluscs.

CgNR1Jb, originally assembled with the early development group, displayed its highest expression in the adult life stages (Figs. 2 and 4), but also showed measurable expression during most of the other life stages. CgNR1Jb and CgNR1Ja, which showed a similar expression profile to CgNR1Jb, are members of the protostomes subfamily group NR1J, a homologous group to the deuterostome subfamily group NR1I. Representatives of the NR1I and NR1J subfamilies have been linked to xenobiotic-sensing [96–101], a mechanism of defence against natural and anthropogenic environmental stressors through which expression of genes involved in detoxification is induced [100, 101]. Research in the bivalve *Scorbicularia plana* demonstrated that the bivalve homolog NR1Jβ is also able to interact with such compounds, suggesting a conserved xenobiotic-sensing mechanism in bivalves [96].

## Conclusion

This study provides a detailed overview of the NR expression dynamics in the Pacific oyster. We have demonstrated that a large variety of NRs are expressed at different respective stages throughout oyster lifetime, ranging from fertilisation, through embryo and larval development, to the point of adulthood. Different NRs cluster together into groups in a non-phylogenetic manner, representing different life events such as early, mid and late development. Differences between sexes were also recognized.

NRs are known to interact with ligands, which makes them vulnerable to exogenous xenobiotic compounds. Therefore, detecting the expression dynamics in different life stages is important in predicting putative functions of NRs and helping to uncover at which life points the Pacific oyster is vulnerable to xenobiotics. Our study on NRs in a molluscan species is therefore an important step towards understanding invertebrate development and for the study of anthropogenic impacts on the environment.

## Methods

### Oyster husbandry

Development studies were adapted from the oyster embryo-larval bioassay protocol [102]. Three independent fertilisations were performed using four female and five male conditioned adult individuals (Guernsey Sea Farm, Guernsey, UK). Artificial seawater (ASW) was prepared several days prior at 34 psu and pH 8.25. Approximately 3,000–4,000 eggs/ml in a total volume of 1 L were fertilized per biological replicate 3 hours post fertilization (hpf) each replicate was diluted 1:20 (to ~150-200 embryos/ml) in ASW to prevent oxygen depletion. Life stages were microscopically assessed and samples were taken of unfertilized eggs (~100,000 eggs/sample) and at 1 hpf, 2 hpf, 3 hpf (~100,000 embryos/sample), 6 hpf, 12 hpf and 27 hpf (3000–6000 embryos/sample) (Fig. 1: 1–7) from the three independent developmental experiments. The experiments were validated by assessing the percentage of dead/abnormally developed D-shelled larvae (ranging from 0 to 15 % abnormal development), which did not exceed the critical rejecting value of 30 %.

Veliger (7 days post fertilisation (dpf)) and pediveliger (15 dpf) larvae stages, as well as adult individuals were also obtained from Guernsey Sea Farm. The larvae were re-suspended in 12 °C artificial seawater for one hour and sampled (Fig. 1: 8–9). Three samples of veliger (1200–2000 larvae/sample) and pediveliger (500–100 larvae/samples), respectively, were taken for further analysis. Three male and three female adult individuals at the beginning of the gametogenesis were shucked, and tissues homogenised and pooled separately by sex (Fig. 1: 10a–10b). Three homogenized pooled tissue samples (~100 mg) for each sex were taken for further analysis. The sex of the individuals was determined through examination of the gonads to verify whether sperm or eggs were present. Only individuals in the early stages of gametogenesis were used.

### RNA extraction and reverse transcription

The RNA was extracted from three biological replicates at each time point (developmental stages: unfertilized eggs, 1 hpf – 27 hpf; veliger larvae (7 dpf); pediveliger (15 dpf; pooled female and adult individuals). Total RNA was extracted using TRI Reagent RNA Isolation Reagent (Sigma-Aldrich), following the manufacturer’s protocol, and genomic DNA was removed with RQ1 RNase-Free DNase (Promega). RNA purity and quantity were determined by ND-1000 spectrophotometer (Nanodrop). For each sample, 900 ng of total RNA, divided into two independent 20 μl reactions (each 450 ng RNA), was converted to cDNA with the ThermoScript RT/PCR System (Invitrogen), using oligo (dT) primers, and then pooled together and further diluted (1:1) with nuclease-free water.

### Primer design and optimisation

Forward and reverse oligonucleotide primers were designed with Primer-Blast at the National Centre for Biotechnology Information (NCBI) [103] to amplify each of the 34 *C. gigas* NRs. Primers were 18–23 nt, with a GC content of 40–60 % and produced predicted amplicons of 100–200 bp (Additional file 3). The primer pairs were optimized by changing final primer concentration, temperature and/or final MgCl_2_ concentration to reach a primer pair efficiency between 90 and 115 %. The efficiency was tested by a dilution series resulting in a standard curve with a slope between 3.0 and 3.55. The efficiency was calculated as follows [104]: Efficiency (E) = 10^(-1/slope)^.

Each primer pair amplification product was verified by sequencing, using a common polymerase chain reaction (PCR) with the GoTaq system (Promega) for amplification and the products were purified with the QIAquick PCR Purification Kit (Qiagen, UK). Sequencing was conducted by Eurofins MWG Operon (Ebersberg, Germany).

### Quantitative RT-PCR

Quantitative RT PCR was performed using the SsoFast EvaGreen Supermix (Bio-Rad), and the reactions were run on a CFX96 Real-Time PCR Detection System (Bio-Rad). For each gene, each of the biological replicates per time point was run in duplicate (technical replicates) on a single plate. 0.5 μl of cDNA dilution was used per 10 μl reaction. The MgCl_2_ concentration of the SsoFast EvaGreen supermix and the primer concentration were adjusted for primer optimisation (Additional file 3). qPCR conditions were as follows: 95 °C for 2 min, 45 cycles of 95 °C for 15 s, 60–63.1 °C for 30 s (primer dependent) and 72 °C for 1 min. A melt curve was run after each PCR (65–95 °C at a temperature transition rate of 0.05 °C/s). For each reaction the melt curves were analysed to verify the specificity of the amplified product, and to confirm that a single PCR product had been amplified. A non-template control was analysed in parallel for each gene as well as a positive control.

### Data analysis

The calculation of the relative expression (RE) for gene transcripts (mRNA copies) of each NR was based on the modified comparative Ct method [104, 105], using the average Ct (avCt_target_) of each biological replicate per time point, corrected for efficiency (E) and compared to the normalisation factor of combined reference genes (NF_refs_). Three housekeeping genes (elongation factor-1 α (EF-1), ribosomal protein S18 (RS18), ribosomal protein L7 (RL7)) were chosen as reference genes (Additional file 1). The normalisation factor of the combined reference gene (NF_refs_) has been determined by the programme geNorm v3 [106], using the Ct values of the reference genes corrected for their efficiency:

$\mathrm{Normalisation}\ \mathrm{Factor}\ \left(\mathrm{N}{\mathrm{F}}_{\mathrm{ref}\mathrm{n}}\right)=\sqrt[\mathrm{n}]{{{\mathrm{E}}_{\mathrm{ref}1}}^{\mathrm{avCtref}1}{{*\mathrm{E}}_{\mathrm{ref}2}}^{\mathrm{avCtref}2}*\dots {{*\mathrm{E}}_{\mathrm{ref}\mathrm{n}}}^{\mathrm{avCtref}\mathrm{n}}}$. Thus, the relative expression was calculated as follows [105]: $\mathsf{Relative}\ \mathsf{expression}\ \left(\mathrm{R}\mathrm{E}\right)=\frac{{\mathrm{NF}}_{\mathrm{refn}}}{{{\mathrm{E}}_{\mathrm{target}}}^{\mathrm{avCttarget}}}$.

Relative expressions of all NRs were statistically analysed using RStudio v0.98.1091 (RStudio, Inc.). The data was transformed (log or sqrt) to normal distributions, tested using a Shapiro-Wilk test, and the expression patterns were analysed with a one-way ANOVA follows by multiple pairwise comparisons with Tukey’s Honestly Significant Difference Test. Cluster analysis was performed to demarcate the expression patterns during all stages using Cluster 3.0 v1.52 [107]. Hierarchical and *k*-means clusters were obtained by logarithmically transformed centred data, by gene, using the Euclidean similarity metric. The hierarchical cluster was visualized using Java TreeView [108]. In addition, to examining gene expression patterns along NR subfamilies and developmental stages excluding and including adult stages, principal component analyses (PCA) were conducted in R version 3.2.4 [109], using prcomp(), a built-in function in the R stats package, and the packages FactorMineR [110] and factoextra [111].

## Additional files

Additional file 1: Gene expression of selected reference genes in *Crassostrea gigas* among different life stages. Three housekeeping genes selected as reference genes (centred Ct values): elongation factor-1α: white bars; ribosomal protein S18: light grey bars; ribosomal protein L7: dark grey bars; mean of all reference genes including significantly different groups: black bars and letters. hpf: hour post fertilisation. dpf: days post fertilisation. E: unfertilised eggs. F: female. M: male. (DOC 43 kb) Additional file 2: Principle component analysis (PCA) of 31 *Crassostrea gigas* nuclear receptor gene expression data across developmental and adult life stages. Scatterplot of the first two PCA components of developmental stages including adult life stages indicating separation of all life stages based upon the variance observed in the expression levels of 31 of the 34 nuclear receptor genes. Principal component 1 (PC1) and 2 (PC2) explain 36 and 25 % of variance, respectively Circles around measurements and colours of measurements representing distinct clustering for all life stages or nuclear receptors: early development (green numbers + letters, black solid line), mid development (red numbers + letters, black dotted lines), late development (blue numbers + letters, black dashed line), adult life stages (yellow letters, grey dashed lines). h: hour post fertilisation; d: days post fertilisation; E: unfertilised eggs; F: female; M: male. (DOC 196 kb) Additional file 3: Primer information used in qPCR analysis. Table of forward and reverse primer sequences for 34 *Crassostrea gigas* nuclear receptors and three reference genes including amplicon length (bp), annealing temperature, final primer concentration and MgCl_2_ concentration, primer efficiency, and accession number of receptor gene. (XLS 41 kb)

## References

[1] Bridgham JT, Eick GN, Larroux C, Deshpande K, Harms MJ, Gauthier MEA, Ortlund EA, Degnan BM, Thornton JW (2010). Protein evolution by molecular tinkering: diversification of the nuclear receptor superfamily from a ligand–dependent ancestor. PLoS Biol.

[2] Bertrand S, Brunet FG, Escriva H, Parmentier G, Laudet V, Robinson-Rechavi M (2004). Evolutionary genomics of nuclear receptors: from twenty–five ancestral genes to derived endocrine systems. Mol Biol Evol.

[3] Laudet V, Hänni C, Coll J, Catzeflis C, Stéhelin D (1992). Evolution of the nuclear receptor gene family. EMBO J.

[4] Laudet V (1997). Evolution of the nuclear receptor superfamily: early diversification from an ancestral orphan receptor. J Mol Endocrinol.

[5] Bruce CM, Campbell MJ. Nuclear Receptors: current concepts and future challenges. Dordrecht: Springer; 2010.

[6] Taneja R (2006). Nuclear receptors in development.

[7] Nuclear Receptors Nomenclature Committee (1999). A unified nomenclature system for the nuclear receptor superfamily. Cell.

[8] Wu W, Niles EG, Hirai H, LoVerde PT (2007). Evolution of a novel subfamily of nuclear receptors with members that each contain two DNA binding domains. BMC Evol Biol.

[9] Germain P, Staels B, Dacquet C, Spedding M, Laudet V (2006). Overview of nomenclature of nuclear receptors. Pharmacol Rev.

[10] Mangelsdorf DJ, Thummel C, Beato M, Herrlich P, Schütz G, Kastner P, Mark M, Chambon P, Evans RM (1995). The nuclear receptor superfamily: the second decade. Cell.

[11] McLachlan JA (2001). Environmental signaling: what embryos and evolution teach us about endocrine disrupting chemicals. Endocr Rev.

[12] Bergman Å, Heindel JJ, Jobling S, Kidd KA, Zoeller RT (2013). The state-of-the-science of endocrine disrupting chemicals - 2012.

[13] Gore AC, Chappell VA, Fenton SE, Flaws JA, Nadal A, Prins GS, Toppari J, Zoeller RT (2015). Executive summary to EDC-2: the endocrine society’s second scientific statement on endocrine-disrupting chemicals. Endocr Rev.

[14] Segner H, Gupta RC (2011). Reproductive and developmental toxicity in fishes. Reproductive and Developmental Toxicology.

[15] Canesi L, Fabbri E (2015). Environmental effects of BPA: focus on aquatic species. Dose Response.

[16] Miyagawa S, Lange A, Hirakawa I, Tohyama S, Ogino Y, Mizutani T, Kagami Y, Kusano T, Ihara M, Tanaka H, Tarazako N, Ohta Y, Katsu Y, Tyler CR, Iguchi T (2014). Differing species responsiveness of estrogenic contaminants in fish is conferred by the ligand binding domain of the estrogen receptor. Environ Sci Technol.

[17] Lange A, Katsu Y, Miyagawa S, Ogino Y, Urushitani H, Kobayashi T, Hirai T, Shears JA, Nagae M, Yamamoto J, Ohnishi Y, Oka T, Tatarazako N, Ohta Y, Tyler CR, Iguchi T (2012). Comparative responsiveness to natural and synthetic estrogens of fish species commonly used in the laboratory and field monitoring. Aquat Toxicol.

[18] Delfosse V, Maire AL, Balaguer P, Bourguet W (2015). A structural perspective on nuclear receptors as targets of environmental compounds. Acta Pharmacol Sin.

[19] Titley-O’Neal CP, Munkittrick KR, Macdonald BA (2011). The effects of organotin on female gastropods. J Environ Monit.

[20] Ruiz JM, Bryan GW, Gibbs PE (1995). Acute and chronic toxicity of tributyltin (TBT) to pediveliger larvae of the bivalve *Scrobicularia plana*. Mar Biol.

[21] Ruiz JM, Bryan GW, Wigham GD, Gibbs PE (1995). Effects of tributyltin (TBT) exposure on the reproduction and embryonic development of the bivalve *Scrobicularia plana*. Mar Envir R.

[22] Inoue S, Oshima Y, Nagai K, Yamamoto T, Go J, Kai N, Honjo T (2004). Effect of maternal exposure to tributyltin on reproduction of the pearl oyster (*Pinctada fucata martensii*). Environ Toxicol Chem.

[23] Park JJ, Shin YK, Hung AAO, Romano N, Cheom YP, Kim JW (2015). Reproductive impairment and intersexuality in *Gomphina veneriformis* (Bivalvia: Veneridae) by the tributyltin compound. Animal Cells System.

[24] Le Maire A, Grimaldi M, Roeckling D, Dagnino S, Vivat-Hannah V, Balaguer P, Bourguet W (2009). Activation of RXR–PPAR heterodimers by organotin environmental endocrine disruptors. EMBO Rep.

[25] Urushitani H, Katsu Y, Ohta Y, Shiraishi H, Iguchi T, Horiguchi T (2011). Cloning and characterization of retinoid X receptor (RXR) isoforms in the rock shell, *Thais clavigera*. Aquat Toxicol.

[26] Grün F, Watanbe H, Zamanian Z, Maeda L, Arima K, Cubacha R, Gardiner DM, Kanno J, Iguchi T, Blumberg B (2006). Endocrine–disrupting organotin compounds are potent inducers of adipogenesis in vertebrates. Mol Endocrinol.

[27] Nishikawa J, Mamiya S, Kanayama T, Nishikawa T, Shiraishi F, Horiguchi T (2004). Involvement of the retinoid X receptor in the development of imposex caused by organotins in gastropods. Environ Sci Technol.

[28] Iguchi T, Katsu Y (2008). Commonality in signaling of endocrine disruption from snail to human. Bioscience.

[29] Vogeler S, Galloway TS, Lyons BP, Bean TP (2014). The nuclear receptor gene family in the Pacific oyster, *Crassostrea gigas*, contains a novel subfamily group. BMC Genomics.

[30] Zhou Q, Zhang J, Fu J, Shi J, Jiang G (2008). Biomonitoring: an appealing tool for assessment of metal pollution in the aquatic ecosystem. Analytical Chimc Acta.

[31] McClellan–Green PD, Matthiessen P (2013). Endocrine disruption in molluscs: processes and testing. Endocrine Disrupters: Hazard Testing and Assessment Methods.

[32] Suárez–Ulloa V, Fernández–Tajes J, Manfrin C, Gerdol M, Venier P, Eirín–López JM (2013). Bivalve Omics: state of the art and potential applications for the biomonitoring of harmful marine compounds. Mar Drugs.

[33] Goodrich RJ, Anton E, Krawetz SA (2013). Isolation mRNA and small noncoding RNAs from human sperm. Methods Mol Biol.

[34] Davidson E (1986). Gene Activity in Early Development.

[35] Tandros W, Lipshitz HD (2009). The maternal–to–zygotic transition: a play in two acts. Development.

[36] Bashirullah A, Halsell SR, Cooperstock RL, Kloc M, Karaiskakis A, Fisher WW, Fu W, Hamilton JK, Etkin LD, Lipshitz HD (1999). Joint action of two RNA degradation pathways controls the timing of maternal transcript elimination at the midblastula transition in *Drosophila melanogaster*. EMBO J.

[37] Bashirullah A, Cooperstock RL, Lipshitz HD (2001). Spatial and temporal control of RNA stability. Proc Natl Acad Sci U S A.

[38] De Renzis S, Elemento O, Tavazoie S, Wieschaus F (2007). Unmasking activation of the zygotic genome using chromosomal deletions in the *Drosophila* embryo. PLoS Biol.

[39] Liang HL, Nien CY, Liu HY, Metzgenstein MM, Kirov N, Rushlow C (2008). The zinc–finger protein Zelda is a key activator of the early zygotic genome in *Drosophila*. Nature Letters.

[40] Benoit B, He CH, Zhang F, Votruba SM, Tadros W, Westwood JT, Smibert CA, Lipshitz HD, Theurkauf WE (2009). An essential role for the RNA–binding protein Smaug during the *Drosophila* maternal–to–zygotic transition. Development.

[41] Zhong W, Sladek FM, Darnell JE (1993). The expression pattern of a *Drosophila* homolog to the mouse transcription factor HNF–4 suggests a determinative role in gut formation. Embo J.

[42] Holewa B, van Pogge Strandmann E, Zapp D, Lorenz P, Ryffel GU (1996). Transcriptional hierarchy in *Xenopus* embryogenesis: HNF4 a maternal factor involved in the developmental activation of the gene encoding the tissue specific transcription factor HNF1 alpha (LFB1). Mech Dev.

[43] Holewa B, Zapp D, Drewes T, Senkel S, Ryffel GU (1997). HNF4beta, a new gene of the HNF4 family with distinct activation and expression profiles in oogenesis and embryogenesis of *Xenopus laevis*. Mol Cell Biol.

[44] Ryffel GU, Lingott A (2000). Distinct promoter elements mediate endodermal and mesodermal expression of the HNF1alpha promoter in transgenic *Xenopus*. Mech Dev.

[45] Nastos A, van Pogge Strandmann E, Weber H, Ryffel GU (1998). The embryonic expression of the tissue–specific transcription factor HNF1alpha in *Xenopus*: rapid activation by HNF4 and delayed induction by mesoderm inducers. Nucleic Acids Res.

[46] Duncan SA, Manova K, Chen WS, Hoodless P, Weinstein DC, Bachvarova RF, Darnell JE (1994). Expression of transcription factor HNF–4 in the extraembryonic endoderm, gut, and nephrogenic tissue of the developing mouse embryo: HNF–4 is a marker for primary endoderm in the implanting blastocyst. Proc Natl Acad Sci U S A.

[47] Chen WS, Manova K, Weinstein DC, Duncan SA, Plump AS, Prezioso VR, Bachvarova RF, Darnell JE (1994). Disruption of the HNF–4 gene, expressed in visceral endoderm, leads to cell death in embryonic ectoderm and impaired gastrulation of mouse embryos. Genes Dev.

[48] Xu J, Tan A, Palli SR (2010). The function of nuclear receptors in regulation of female reproduction and embryogenesis in the red flour beetle, *Tribolium castaneum*. J Insect Physiol.

[49] Ables ET, Bois KE, Garcia CA, Drummond-Barbosa D (2015). Ecdysone response gene E78 controls ovarian germline stem cell niche formation and follicle survival in *Drosophila*. Dev Biol.

[50] Gosling E (2004). Bivalve Molluscs: Biology, Ecology and Culture. Reprint.

[51] Kakoi S, Kin K, Miyazaki K, Wada H (2008). Early development of Japanese spiny oyster (*Saccostrea kegaki*). Characterization of some genetic markers. Zoolog Science.

[52] Creton R, Zwaan G, Dohmen R (1993). Specific developmental defects in molluscs after treatment with retinoic acid during gastrulation. Dev Growth Differ.

[53] Carter CJ, Farrar N, Carlone RI, Spencer GE (2010). Developmental expression of a molluscan RXR and evidence for its novel, nongenomic role in growth cone guidance. Dev Biol.

[54] Carter CJ, Rand C, Mohammad I, Lepp A, Vesprini N, Wiebe O, Carlone R, Spencer GE (2015). Expression of a retinoic acid receptor (RAR)–like protein in the embryonic and adult nervous system of a protostome species. J Exp Zool.

[55] Bouton D, Escriva H, de Mendonca RL, Glineur C, Bertin B, Noël C, Robinson–Rechavi M, de Groot A, Cornette J, Laudet V, Pierce RJ (2005). A conservated retinoid X receptor (RXR) from mollusc *Biomphalaria glabrata* transactivates transcription in the presence of retinoids. J Mol Endocrinol.

[56] Thain JE, Waldock MJ (1986). The impact of tributyl tin (TBT) antifouling paints on molluscan fisheries. Wat Sci Tech.

[57] Salazar MH, Salazar SM, Champ MA, Seligman PF (1996). Mussel as bioindicators: effects of TBT on survival, bioaccumulation, and growth under natural conditions. Organotin: Environmental Fate and Effects.

[58] Thain JE (1986). Toxicity of TBT to Bivalves: effects on reproduction growth and survival. Proceedings of the Oceans ’86 Organotin Symposium, Vol. 4: 23-25 Sept. 1986.

[59] Mangelsdorf DJ, Evans RM (1995). The RXR heterodimers and orphans receptors. Cell.

[60] Szantos A, Narkar V, Shen Q, Uray IP, Davis PJA, Nagy L (2004). Retinoid X receptors: X–ploring their (patho)physiological functions. Cell Death Diff.

[61] Urushitani H, Katsu Y, Ohta Y, Shiraishi H, Iguchi T, Horiguchi T (2013). Cloning and characterization of the retinoic acid receptor–like protein in the rock shell, *Thais clavigera*. Aquat Toxicol.

[62] Gutierrez–Mezariegos J, Nadendla EK, Lima D, Pierzchalski K, Jones JW, Kane M, Nishikawa JI, Hiromori Y, Nakanishi T, Santos MM, Castro FC, Bourguet W, Schubert M, Laudet V (2014). A mollusc retinoid acid receptor (RAR) ortholog sheds light on the evolution of ligand binding. Endocrinology.

[63] André A, Ruivo R, Gesto M, Castro LF, Santos MM (2014). Retinoid metabolism in invertebrates: when evolution meets endocrine disruption. Gen Comp Endocrinol.

[64] Niederreither K, Dolle P (2008). Retinoic acid in development towards an integrated view. Nature.

[65] Kaur S, Jobling S, Jones CS, Noble LR, Routledge EJ, Lockyer AE (2015). The nuclear receptor of *Biomphalaria glabrata* and *Lottia gigantea*: implications for developing new model organisms. PLoS One.

[66] Michalik L, Desvergne B, Dreyer C, Gavillet M, Laurini RN, Wahli W (2002). PPAR expression and function during vertebrate development. Int J Dev Biol.

[67] Rotman N, Guex N, Gouranton E, Wahli W (2013). PPARβ Interprets a chromatin signature of pluripotency to promote embryonic differentiation at gastrulation. PLoS One.

[68] Kanayama T, Kobayashi N, Mamiya S, Nakanishi T, Nishikawa J (2005). Organotin compounds promote adipocyte differentiation as agonists of the peroxisome proliferator-activated receptor gamma/retinoid X receptor pathway. Mol Pharmacol.

[69] Harada S, Hiromori Y, Nakamura S, Kawahara K, Fukakusa S, Maruno T, Noda M, Uchiyama S, Fukui K, Nishikawa J, Nagase H, Koboyashi Y, Yoshida T, Ohkubo T, Nakanishi T (2015). Structural basis for PPARγ transactivation by endocrine-disrupting organotin compounds. Scientific Reports.

[70] Darras VM, van Herck SLJ, Heijlen M, de Groef B. Thyroid hormone receptors in two model species for vertebrate embryonic development: chicken and zebrafish. J Thyroid Res. 2011;402320. 10.4061/2011/402320 PMC3134294 21760979

[71] Huang W, Xu F, Qu T, Li L, Que H, Zhang G (2015). Iodothyronine deiodinase gene analysis of the Pacific oyster *Crassostrea gigas* reveals possible conservation of thyroid hormone feedback regulation mechanisms in mollusks. Chinese J Ocean Limnol.

[72] Moriyama K, Tagami T, Akamizu T, Usui T, Saijo M, Kanamoto N, Hataya Y, Shimatsu A, Kuzuya H, Nakao K (2002). Thyroid hormone action is disrupted by bisphenol A as an antagonist. J Clin Endocrinol Metab.

[73] Zoeller RT, Bansal R, Parris C (2005). Bisphenol-A, an environmental contaminant that acts as a thyroid hormone receptor antagonist in vitro, increases serum thyroxine, and alters RC3/neurogranin expression in the developing rat brain. Endocrinology.

[74] Ellis I, Kempf SC (2011). Characterization of the central nervous system and various peripheral innervations during larval development of the oyster *Crassostrea virgonica*. Invert Biol.

[75] Helm MM, Bourne N, Lovatelli A (2004). Hatchery Culture of Bivalve. A Practical Manual.

[76] Riddiford LM, Truman JW, Mirth CK, Shen Y (2010). A role for juvenile hormone in the prepupal development of *Drosophila melanogaster*. Development.

[77] Thummel CS (2001). Molecular mechanisms of developmental timing in *C. elegans* and *drosophila*. Dev Cell.

[78] Fahrbach SE, Smagghe G, Velarde RA (2012). Insect nuclear receptors. Annu Rev Entomol.

[79] Laguerre M, Veenstra JA (2010). Ecdysone receptor homologs from mollusks, leeches and a polychaete worm. FEBS Lett.

[80] Bardet PL, Laudet V, Vanacker JM (2006). Studying non–mammalian models? Not a fool’s ERRand!. Trends Endocrinol Metab.

[81] Palanker L, Necakov AS, Sampson HM, Ni R, Hu C, Thummel CS, Krause HM (2006). Dynamic regulation of *Drosophila* nuclear receptor activity in vivo. Development.

[82] Tennessen JM, Baker KD, Lam G, Evans J, Thummel CS (2011). The Drosophila estrogen–related receptor directs a metabolic switch that supports developmental growth. Cell Metabol.

[83] Strobl–Mazzulla PH, Lethimonier C, Gueguen MM, Karube M, Fernandino JI, Yoshizaki G, Patino R, Strüssmann CA, Kah O, Somozoa GM (2008). Brain aromatase (Cyp19A2) and estrogen receptors, in larvae and adult pejerrey fish *Odontesthes bonariensis*: neuroanatomical and functional relations. Gen Comp Endocrinol.

[84] Fröhlicher M, Liedtke A, Groh K, Lopez–Schier H, Neuhauss SCF, Segner H, Eggen RIL (2009). Estrogen receptor subtype β2 is involved in neuromast development in zebrafish (*Danio rerio*) larvae. Dev Biol.

[85] Bannister R, Beresford N, May D, Routledge EJ, Jobling S, Rand–Weaver M (2007). Novel estrogen receptor–related transcripts in *Marisa cornuarietis*; a freshwater snail with reported sensitivity to estrogenic chemicals. Environ Sci Technol.

[86] Bannister R, Beresford N, Granger DW, Pounds NA, Rand-Weaver M, White R, Jobling S, Routledge EJ (2013). No substantial changes in estrogen receptor and estrogen–related receptor orthologues gene transcription in Marisa cornuarietis exposed to estrogenic chemicals. Aquat Toxicol.

[87] Matsumoto T, Nakamura AM, Mori K, Akiyama I, Hirose H, Takahashi Y (2007). Oyster estrogen receptor: cDNA cloning and immunolocalization. Gen Comp Endocrinol.

[88] Guo X, Hedgecock D, Hershberger WK, Cooper K, Allen SK (1998). Genetic determinations of podrandric sex in the Pacific oyster, *Crassostrea gigas* Thunberg. Evolution.

[89] Carmi I, Kopczynski JB, Meyer BJ (1998). The nuclear hormone receptor SEX–1 is an X–chromosome signal that determines nematode sex. Nature.

[90] Norris DO, Lopez KH. Hormones and reproduction of vertebrates. 1^st^ ed. Burlington: Elsevier: 2013.

[91] Kajiwara M, Kuraku S, Kurokawa T, Kato K, Toda S, Hirose H, Takahashi S, Shibata Y, Iguchi T, Matsumoto T, Miyata T, Miura T, Takahashi Y (2006). Tissue preferential expression of estrogen receptor gene in the marine snail, *Thais clavigera*. Gen Comp Endocrinol.

[92] Waldock MJ, Thain JE (1983). Shell thickening in *Crassostrea gigas*: organotin antifouling or sediment induced?. Mar Pollut Bull.

[93] Higuera-Ruiz R, Elorza J (2011). Shell thickening and chambering in the oyster *Crassostrea gigas*: natural and anthropogenic influence of tributyltin contamination. Environ Technol.

[94] Nakanishi T (2007). Potential toxicity of organotin compounds via nuclear receptor signaling in mammals. J Health Sci.

[95] Pascoal S, Carvalho G, Vasieva O, Hughes R, Cossins A, Fang Y, Ashelford K, Olohan L, Barroso C, Mendo S, Creer S (2013). Transcriptomics and in vivo tests reveal novel mechanisms underlying endocrine disruption in an ecological sentinel, *Nucella lapillus*. Mol Ecol.

[96] Cruzeiro C, Lopes-Marques M, Ruivo R, Rodrigues-Oliveira N, Santos MM, Rocha MJ, Rocha E, Castro LF (2016). A mollusk VDR/PXR/CAR-like (NR1J) nuclear receptor provides insight into ancient detoxification mechanisms. Aquat Toxicol.

[97] Karimullina E, Li Y, Ginjupalli G, Baldwin WS (2012). *Daphnia* HR96 is a promiscuous xenobiotic and endobiotic nuclear receptor. Aquat Toxicol.

[98] King-Jones K, Horner MA, Lam G, Thummel CS (2006). The DHR96 nuclear receptor regulates xenobiotic responses in *Drosophila*. Cell Metab.

[99] Timsit YE, Negishi M (2007). CAR and PXR: the xenobiotic-sensing receptors. Steroids.

[100] Richter I, Fiddler AE (2014). Marine invertebrate xenobiotic-activated nuclear receptors: their application as sensor elements in high-throughput bioassay for marine bioactive compounds. Mar Drugs.

[101] Prakash C, Zuniga B, Song CS, Jiang S, Cropper J, Park S;Chatterjee B. Nuclear receptors in drug metabolism, drug response and drug interactions. Nucl Receptor Res. 2015; doi: 10.11131/2015/101178. 10.11131/2015/101178 PMC4963026 27478824

[102] Leverett D, Thain J. Oyster embryo–larval bioassay (Revised). In: Keizer PD, editor. ICES Techniques in Marine Environmental Sciemce No. 54. Denmark: International Council for the Exploration of the Sea; 2013. pp. 34.

[103] Ye J, Coulouris G, Zaretskaya I, Cutcutache I, Rozen S, Madden T (2012). Primer–BLAST: a tool to design target–specific primers for polymerase chain reaction. BMC Bioinformatics.

[104] Pfaffl MW (2001). A new mathematical model for relative quantification in real–time RT–PCR. Nucleic Acids Res.

[105] Filby AL, Tyler CR (2005). Molecular characterization of estrogen receptors 1, 2a, and 2b and their tissue and ontogenetic expression profiles in fathead minnow (Pimephales promelas). Biol Reprod.

[106] Vandesompele J, De Preter K, Pattyn F, Poppe B, Van Roy N, De Paepe A, Speleman F (2002). Accurate normalisation of real–time quantitative RT–PCR data by geometric averaging of multiple internal control genes. Genome Biol.

[107] De Hoon MJL, Imoto S, Nolan J, Miyano S (2004). Open source clustering software. Bioinformatics.

[108] Saldanha AJ (2004). Java Treeview – extensible visualization of microarray data. Bioinformatics.

[109] R Development Core Team (2008). R: A Language and Environment for Statistical Computing.

[110] Lê S, Josse J, Husson F (2008). FactoMine R: an R package for multivariate analysis. J Stat Soft.

[111] Factoextra. http://www.sthda.com/english/. Accessed 3 May 2016.

