# Supplementary figures and images for: Dynamics of nuclear receptor gene expression during Pacific oyster development
Source: BMC Dev Biol. 2016 Sep 29;16:33. doi: 10.1186/s12861-016-0129-6 (PMC5041327; doi:10.1186/s12861-016-0129-6)

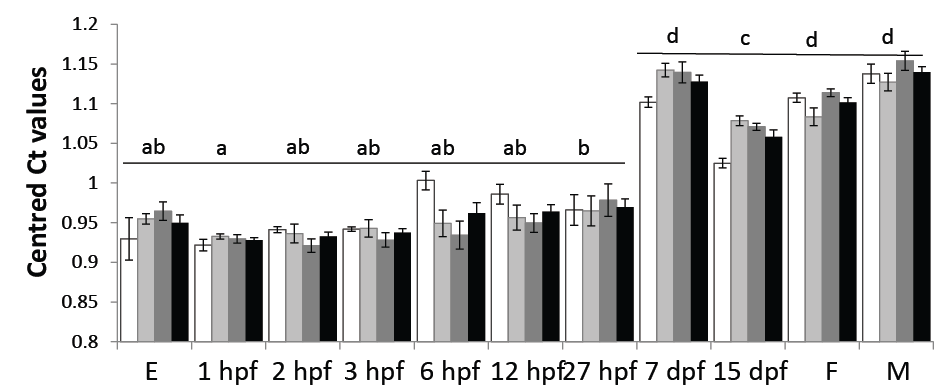

Supplement: Additional file 1: — Gene expression of selected reference genes in Crassostrea gigas among different life stages. Three housekeeping genes selected as reference genes (centred Ct values): elongation factor-1α: white bars; ribosomal protein S18: light grey bars; ribosomal protein L7: dark grey bars; mean of all reference genes including significantly different groups: black bars and letters. hpf: hour post fertilisation. dpf: days post fertilisation. E: unfertilised eggs. F: female. M: male. (DOC 43 kb) [file 12861_2016_129_MOESM1_ESM.doc]

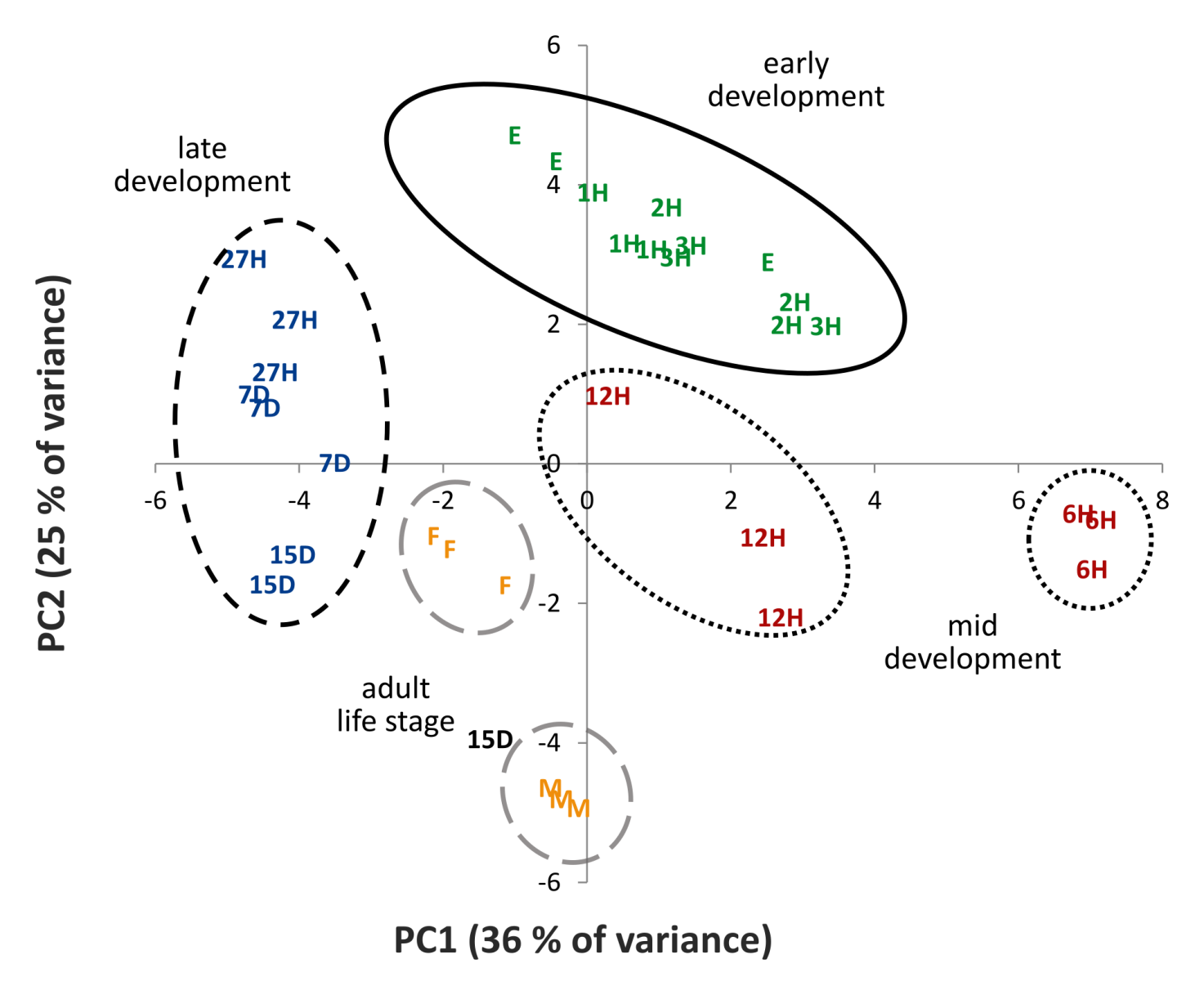

Supplement: Additional file 2: — Principle component analysis (PCA) of 31 Crassostrea gigas nuclear receptor gene expression data across developmental and adult life stages. Scatterplot of the first two PCA components of developmental stages including adult life stages indicating separation of all life stages based upon the variance observed in the expression levels of 31 of the 34 nuclear receptor genes. Principal component 1 (PC1) and 2 (PC2) explain 36 and 25 % of variance, respectively Circles around measurements and colours of measurements representing distinct clustering for all life stages or nuclear receptors: early development (green numbers + letters, black solid line), mid development (red numbers + letters, black dotted lines), late development (blue numbers + letters, black dashed line), adult life stages (yellow letters, grey dashed lines). h: hour post fertilisation; d: days post fertilisation; E: unfertilised eggs; F: female; M: male. (DOC 196 kb) [file 12861_2016_129_MOESM2_ESM.doc]
